# CT characteristics for predicting prognosis of gastric cancer with synchronous peritoneal metastasis

**DOI:** 10.3389/fonc.2022.1061806

**Published:** 2023-01-11

**Authors:** Jiazheng Li, Lin Cong, Xuefeng Sun, Xiaoting Li, Yang Chen, Jieyuan Cai, Meng He, Xiaotian Zhang, Lei Tang

**Affiliations:** ^1^ Department of Radiology, Key Laboratory of Carcinogenesis and Translational Research (Ministry of Education), Peking University Cancer Hospital and Institute, Beijing, China; ^2^ Department of Gastrointestinal Oncology, Key Laboratory of Carcinogenesis and Translational Research (Ministry of Education), Peking University Cancer Hospital and Institute, Beijing, China; ^3^ Department of Radiology, The Affiliated Children's Hospital, Capital Institute of Pediatrics, Beijing, China

**Keywords:** gastric neoplasm, computed tomography, peritoneal metastasis, survival analysis, palliative chemotherapy

## Abstract

**Introduction:**

To explore the CT characteristics for the prediction of long term survival in gastric cancer patients with synchronous peritoneal metastasis (PM).

**Materials and methods:**

Sixty-six patients diagnosed as gastric cancer with synchronous peritoneum metastasis were enrolled in this retrospective study. Ten anatomic peritoneal regions were evaluated to check for the signs of PM on CT. One positive area equaled one score. The CT characteristic-based PM score (CT-PMS) was the sum of the total points assigned to all 10 regions, with a range of 0–10. The triple tract dilatation (TTD) sign caused by peritoneal metastasis, the presence of extensive lymph node metastasis (ELM), and the grade of ascites were recorded. The overall survival (OS) was used as the prognostic indicator. The performance of the CT characteristics was assessed by the Kaplan–Meier analysis and Cox proportional hazards model, while its reproducibility was evaluated by Kappa statistic and weighted Kappa statistic.

**Results:**

Patients with a CT-PMS of 3–10 had significantly poorer OS (*P* = .02). Patients with either the presence of TTD sign, or ELM had a trend toward unfavorable OS (both *P* = .07), and when CT-PMS of 3–10 was detected simultaneously, the survival was further reduced (*P* = .00 for TTD sign; *P* = .01 for ELM). The grade of ascites failed to show a significant correlation with OS. The interobserver reproducibility for assessing the CT-PMS, the presence of TTD sign, the presence of ELM, and the grade of ascites had a substantial to almost perfect agreement.

**Conclusion:**

The prognosis of gastric cancer patients with PM has a correlation with the extent of metastasis dissemination on baseline CT. A CT-PMS of 3–10 is associated with a worse prognosis than that of 0–2. The presence of TTD sign and ELM may help further select patients with extraordinarily poor prognoses.

## Introduction

Approximately 27% of gastric cancer patients have synchronous peritoneal metastases (PM) at initial diagnosis (1). Chemotherapy with palliative intent is less encouraging in 33.3–45.9% of those patients, who experienced disease progression in eight weeks or faced serious adverse events (2). The survival period of these patients is correlated with the severe extent of PM, and the patients with a high PM burden detected during the operation or diagnostic laparoscopy have worse survival than those with a low PM burden (3–6). The peritoneal cancer index (PCI) is a commonly used criterion in clinical practice for the risk assessment of PM based on laparoscopy findings (7). Some studies have verified the efficiency of this criterion for survival prediction (8, 9). However, diagnostic laparoscopy is invasive, which inhibits its clinical application to a certain extent (10).

Computed tomography (CT) is one of the preferred imaging modalities for evaluating PM in patients with gastric cancer, because it affords various advantages, such as noninvasive operation, multiphasic contrast-enhanced images, window adjustment technique, and multiplanar reformatted (MPR) images (11, 12). The typical signs of PM on CT include a smudged appearance, multiple fibrosis strands, nodules, and omental cake, with a positive predictive value that ranges within 75%–83%, demonstrating the auxiliary role of CT in managing patients with PM (13–17).

Several studies have reported the prognostic value of the extent of PM determined by CT (18–20). However, these studies have mainly focused on colorectal and ovarian cancer (18–20). To the best of our knowledge, the prognosis prediction of PM-positive gastric cancer *via* pretreatment CT characteristics has yet to be investigated. Therefore, the purpose of this study is to explore CT features that have an impact on the overall survival (OS), so as to identify patients who cannot benefit from palliative chemotherapy, and thus refine patient selection before treatment.

## Materials and methods

### Patients

This retrospective study was approved by the Institutional Review Board of our hospital (No.2020KT121). The requirement of informed consent was waived off.

The present study enrolled 111 consecutive gastric cancer patients with synchronous PM, who were admitted to our institution and treated with systemic chemotherapy between September 2009 and December 2018. The inclusion criteria were as follows: (i) histopathologically confirmed gastric adenocarcinoma; (ii) availability of complete medical records; (iii) synchronous PM diagnosed by baseline CT or diagnostic laparoscopy and cytology (21); and (iv) patients treated with systemic chemotherapy. The exclusion criteria were as follows: (i) unavailable baseline abdominal and pelvic CT (*n* = 19); (ii) baseline CT performed >30 days before chemotherapy (*n* = 9); (iii) any abdominal invasive procedures performed before baseline CT (the peritoneal scar caused by invasive examinations may mimic the PM, *n* = 11); (iv) for patients diagnosed by diagnostic laparoscopy, CT was performed beyond 30 days before the laparoscopy (*n* = 2); (v) multiple primary cancers (*n* = 2); (vi) liver cirrhosis (*n* = 1); and (vii) previous abdominal inflammatory diseases (*n* = 1). Finally, 66 patients were selected for further analyses. Among them, 57 patients were diagnosed by baseline CT, while the remaining nine were diagnosed by diagnostic laparoscopy. The primary clinical and pathological features of these patients, which included their age, gender, ECOG PS, histology type, degree of differentiation, chemotherapy regimens, the location of the primary tumor, and the Borrmann type, were collected from their medical records (**Table 1**).

**Table 1:** Univariate analysis of prognostic factors that affect the overall survival.

Variables	Total (n=66)	Median survival (IQR) (days)	HR (95% CI)	*P-*value
Age (years), n (%)			0.87 (0.53–1.42)	0.58
<57	30 (45.45%)	323 (121–416)		
≥57	36 (54.55%)	292 (179–476)		
Gender, n (%)			1.17 (0.72–1.91)	0.52
Male	35 (53.03%)	302 (157–406)		
Female	31 (46.97%)	356 (179–460)		
ECOG PS, n (%)			1.33 (0.81–2.17)	0.26
0	34 (51.52%)	373 (212–531)		
1+	32 (48.48%)	270 (140–375)		
Histology type, n (%)				
Diffuse	39 (59.10%)	292 (134–392)	[Reference]	
Intestinal	12 (18.18%)	315 (150–546)	0.54 (0.27–1.08)	0.08
Mixed	11 (16.67%)	396 (257–571)	0.61 (0.31–1.20)	0.15
Unknown	4 (6.06%)	335 (278–373)	0.85 (0.30–2.39)	0.76
Differentiation, n (%)			1.77 (1.00–3.13)	0.05
Well/Moderate	18 (27.27%)	390 (174–647)		
Poor	48 (72.73%)	292 (161–392)		
Chemotherapy regimens, n (%)			1.07 (0.62–1.83)	0.81
SOX	19 (28.79%)	335 (252–476)		
NON-SOX	47 (71.21%)	302 (150–456)		
Location of primary tumor, n (%)			0.85 (0.50–1.45)	0.55
Upper and middle	44 (66.67%)	338 (150–424)		
Lower	22 (33.33%)	270 (174–546)		
Bormann type, n (%)			0.92 (0.55–1.54)	0.74
Type 1–3	42(63.64%)	292 (174–480)		
Type 4	24 (36.36%)	323 (140–416)		
CT-PMS, (median [IQR])	2 (1, 5)	315 (147–456)	1.10 (1.00–1.21)	0.049
Classified CT-PMS			1.84 (1.10–3.07)	0.02
0–2	39 (59.09%)	338 (255–546)		
3–10	27 (40.91%)	258 (100–390)		
Triple tract dilation sign				
Presence	8 (12.12%)	97 (73–292)	1.97 (0.93–4.18)	0.08
Absence	58 (87.88%)	335 (186–460)		
Extensive lymph node metastasis			1.67 (0.96–2.90)	0.07
Presence	18 (27.27%)	252 (104–390)		
Absence	48 (72.73%)	340 (186–476)		
Ascites				
0	10 (15.15%)	315 (157–531)	[Reference]	
1	26 (39.39%)	323 (255–460)	0.92 (0.44–1.92)	0.82
2	23 (34.85%)	270 (161–390)	1.45 (0.68–3.10)	0.33
3	7 (10.61%)	364 (91–690)	0.67 (0.24–1.88)	0.45

HR, hazard ratio; CI, confidence interval; CT-PMS, CT characteristic-based peritoneal metastasis score.

The posttreatment follow-up of the patients was performed every three months in the hospital, outpatient clinic, or *via* telephone. These follow-ups lasted until the death of the patients or the cut-off date (March 22, 2021), whichever was earlier. The outcome of the present study was the OS, which was defined as the time from the diagnosis of PM to the death of the patients or their last follow-up.

### Image acquisition

All patients underwent abdominal and pelvic contrast-enhanced CT examinations, using either the LightSpeed 64 VCT (GE Medical Systems, Milwaukee, WI, USA) or Discovery CT750 HD scanner (GE Medical Systems), after fasting for more than 6 h. To reduce gastrointestinal motility, 10 mg of anisodamine (654-2; Hangzhou Minsheng Pharma, China) was intramuscularly administered 15–20 min prior to the CT examination. Next, 6 g of gas-producing crystals were orally administered with 10 mL of warm water shortly before the CT examination to distend the stomach. The patients were scanned in the supine position. The scan range was from the diaphragmatic dome to the lower margin of the pubis. The following imaging parameters were used: peak tube voltage, 120 kVp; tube current, automatic; collimation thickness, 64.000×0.625 mm; helical pitch, 0.984:1.000, and reconstructed thickness, 5 mm. A nonionic contrast material was injected through the antecubital vein at a rate of 3.5 mL/s (1.5 mL/kg of body weight; iohexol: 300 mg I/mL; Omnipaque, GE Healthcare). The arterial and venous phase scans were performed at 40 s and 70 s after the contrast media injection. The multiplanar reconstruction (MPR) images were obtained with a slice thickness of 5 mm.

### Image interpretation

Two radiologists (doctor A and doctor B, with 15 and 4 years of experience in gastric cancer imaging, respectively), who were blinded to the prognosis data of patients, independently reassessed the baseline multiplanar CT images of all patients to evaluate the following CT characteristics and assess interobserver reproducibility. Finally, Any discrepancy was resolved through a third radiologist (doctor C, with 20 years of experience in gastric cancer imaging).

**Figure 1:**
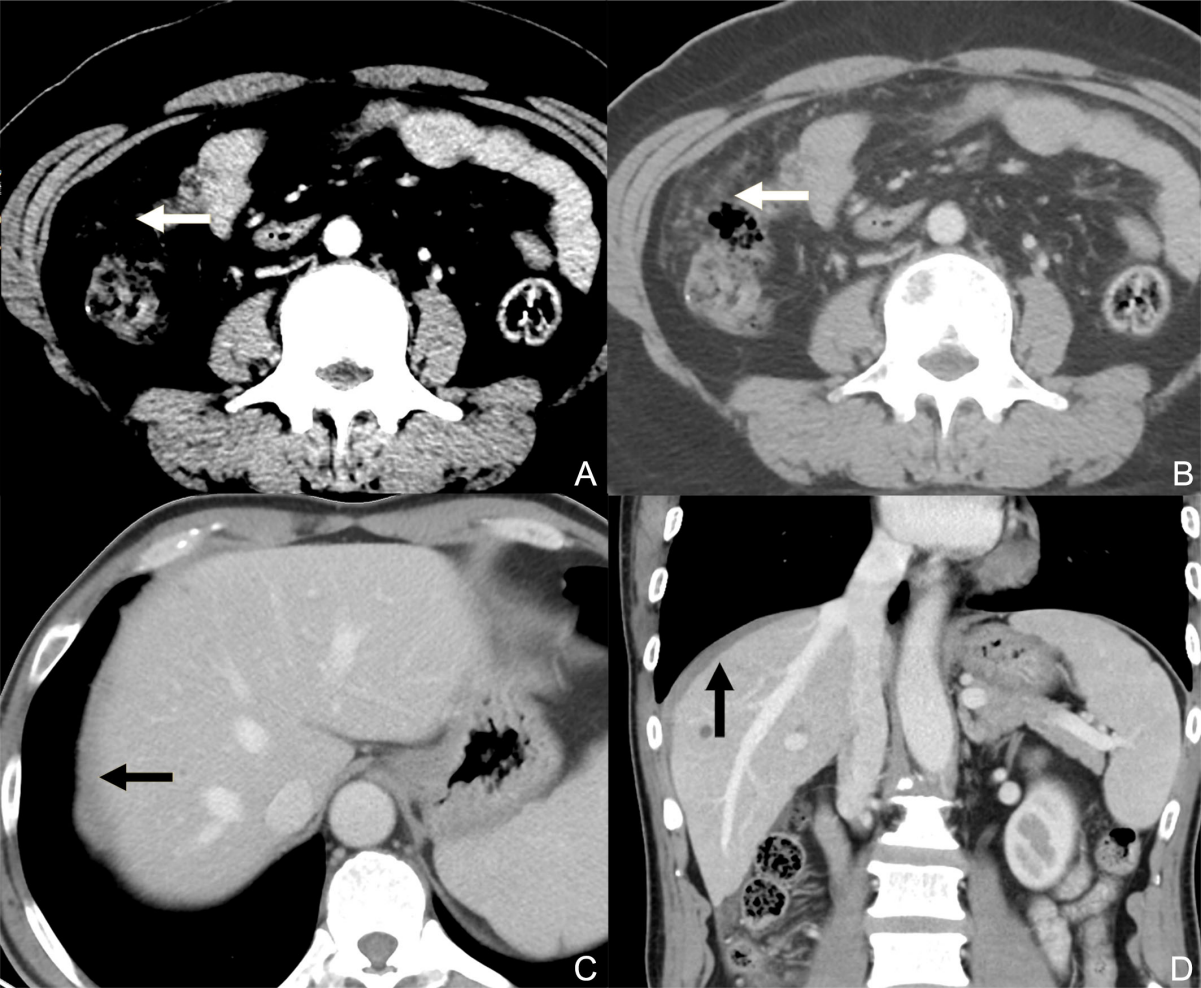
Contrast-enhanced CT axial plane with a narrowed window width **(A)** does not reveal peritoneal metastasis, while that with a wide window width **(B)** clearly reveals discrete nodules on the greater omentum. Contrast-enhanced CT axial plane **(C)** presents the equivocal thickening of the superior parietal peritoneum (black arrow) caused by the partial volume effect. In the sagittal plane **(D)**, the involvement of the superior parietal peritoneum can be better observed as an enhanced diffuse thickening (black arrow, compared with the contralateral superior parietal peritoneum).

**Figure 2:**
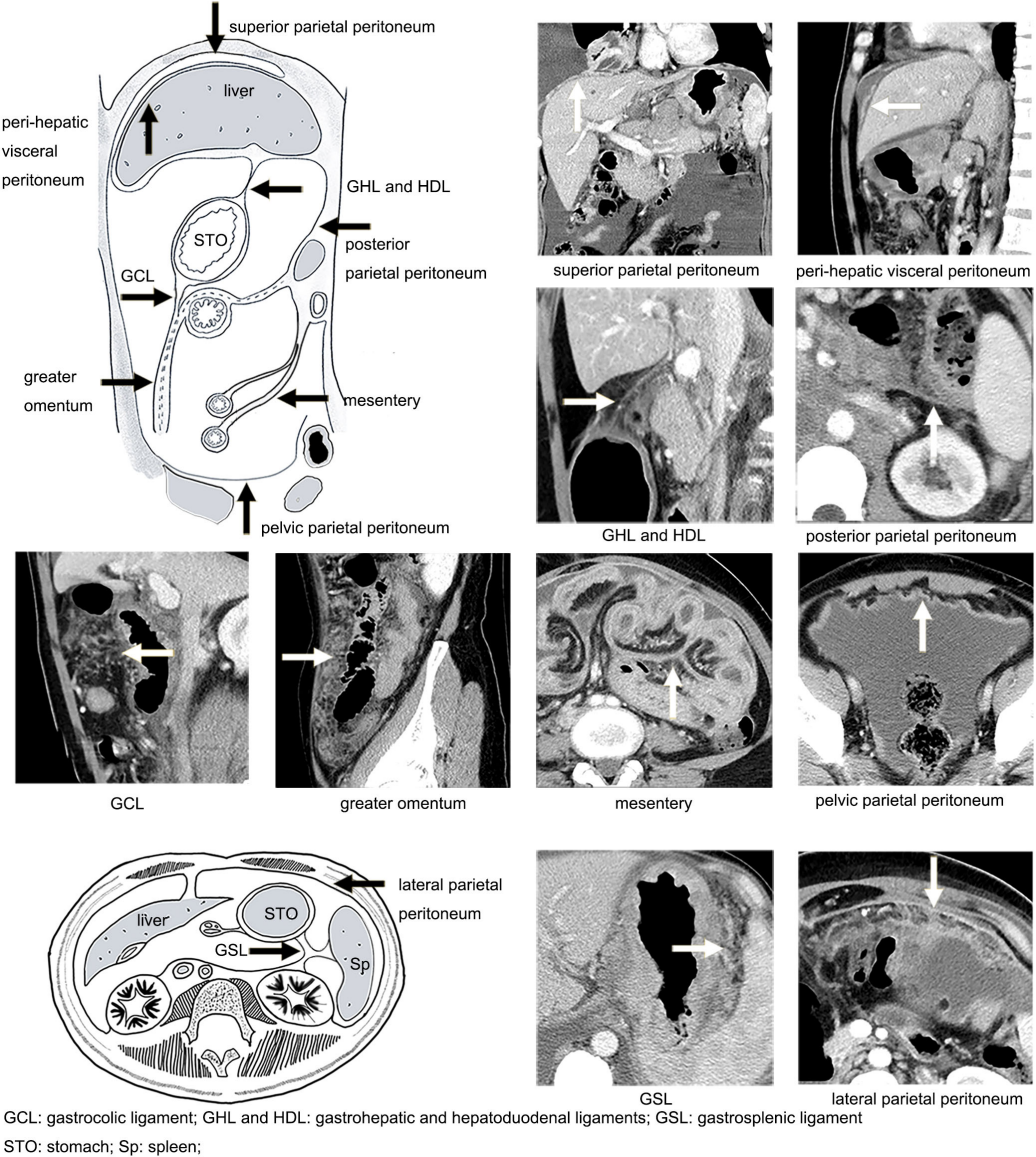
Illustration of the distribution of the peritoneum (black arrow) and manifestations of peritoneal metastasis on CT (white arrow).

The images were reviewed with the dynamic adjustment of the window width and level on Picture Archiving and Communication Systems (PACS) workstations. The wide window width was adjusted to display the tiny grainy background noise of fat tissues to highlight the PM signs (22) (**Figures 1A, B**). A combination of MPR images (axial, coronal, and sagittal planes) was employed to precisely locate the peritoneal regions (**Figures 1C, D**). The following peritoneal regions were evaluated (**Figure 2**) (11, 23, 24): (1) gastrosplenic ligament (GSL); (2) gastrohepatic and hepatoduodenal ligaments (GHL and HDL); (3) gastrocolic ligament (GCL); (4) perihepatic visceral peritoneum; (5) mesentery; (6) greater omentum; (7) superior parietal peritoneum; (8) posterior parietal peritoneum; (9) lateral parietal peritoneum; and (10) pelvic parietal peritoneum. For the score, 1 point was given for the presence of typical PM signs on one peritoneal region, and 0 points were given for its absence. The points obtained from all 10 regions were accumulated to determine the total point for one patient. Thus, the CT-PMS was quantified within the range of 0–10. A CT-PMS of 10 indicated that all 10 peritoneal regions had a typical metastasis sign, while that of 0 indicated the absence of metastasis signs in all peritoneal regions. The typical PM signs on CT included diffuse fibrosis strands, peritoneal nodules, omental cake on the visceral peritoneum, and focal or diffuse thickening with enhancement along the parietal peritoneum (**Table 2**; **Figure 2**) (13–17).

**Table 2:** CT manifestations of peritoneal metastasis (**Figure 2**) (14, 19, 24–26).

Region	Course	Metastasis manifestations
GSL	Connects the greater curvature of the proximal gastric body to the splenic hilum	Smudged appearance, discrete nodules, omental caking
GHL and HDL	Connects the proximal duodenum and lesser curvature of the stomach to the inferior surface of the liver	Smudged appearance
GCL	Connects the greater curvature of the stomach to the transverse colon	Discrete nodules, omental caking
Perihepatic visceral peritoneum	Visceral peritoneum covering the liver	Nodular or diffuse thickening with enhancement
Mesentery	Suspends the jejunum and ileum from the posterior wall of the abdominal cavity	Anomalous fixation of the small intestine, “pleated” or “stellate” appearance
Greater omentum	The GCL extends inferiorly to become a fatty apron-like structure	Smudged appearance, discrete nodules, omental caking
Superior parietal peritoneum	Parietal peritoneum undersurface of the hemidiaphragms	Nodular or diffuse thickening with enhancement
Posterior parietal peritoneum	Parietal peritoneum lines the anterior surface of the retroperitoneum	Nodular or diffuse thickening with enhancement
Lateral parietal peritoneum	Parietal peritoneum lines the lateral abdominal wall	Nodular or diffuse thickening with enhancement
Pelvic parietal peritoneum	Parietal peritoneum lines the pelvic wall and pelvic organ	Nodular or diffuse thickening with enhancement

GSL, gastrosplenic ligament; GHL, gastrohepatic ligament; HDL, hepatoduodenal ligament; GCL, gastrocolic ligament.

**Figure 3:**
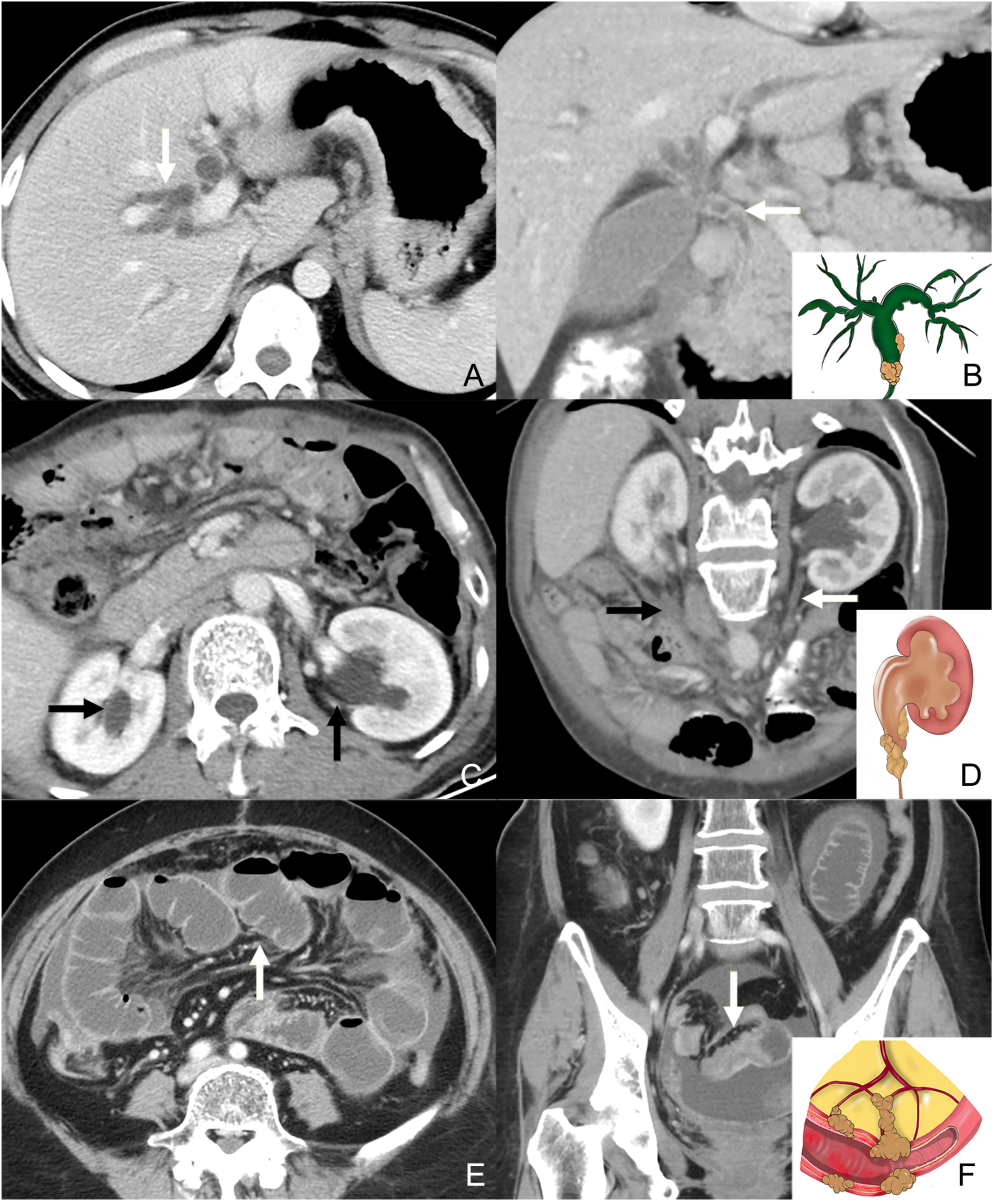
Contrast-enhanced CT axial plane **(A)** and coronal plane (**B**: the same patients with **A**) shows the intrahepatic bile duct dilatation (white arrow in A) caused by the invasion of the common bile duct (white arrow in B). The illustration in the bottom right corner of **(B)** shows the tumor infiltrating the bile duct and dilatation of the proximal canal. Contrast-enhanced CT axial plane **(C)** shows the dilatation of the bilateral urothelial tract (black arrow in C). Contrast-enhanced CT oblique coronal plane (**D**, the same patients with **C**) shows the thickness and enhancement of the left ureter. Note the metastasis on the right side of posterior parietal peritoneum (black arrow). The illustration in the bottom right corner of (**D** shows the tumor infiltrating the bowel wall and dilatation of the proximal canal. Contrast-enhanced CT axial plane **(E)** shows the dilatation of the intestine. Contrast-enhanced CT coronal plane (**F**, the same patients with **E**) shows the thickness and enhancement of the distal intestine. The illustration in the bottom right corner of **(F)** shows the tumor infiltrating the bowel wall and dilatation of the proximal canal.

A number of studies have reported that tumor implants to specific peritoneal sites may narrow or even occlude the nearby cavity organs and cause obstruction of the proximal canal, including biliary obstruction (through the PM of the GHL and HDL), and urothelial obstruction (through the PM of posterior parietal peritoneum) (17, 25, 26). Bowel obstruction included either intestine infiltrated by mesentery PM, or colon infiltrated by GCL and greater omentum PM (26, 27). The presence of obstruction of the bile duct, bilateral urothelial tract, and bowel was evaluated (**Figure 3**). For the bile duct and bilateral urothelial tract, obstruction caused by tumor infiltration may manifest as a focal or diffuse thickening and hyperenhancement of the tract wall, along with the dilatation of the proximal canal. Bowel obstruction included mechanical obstruction, which presents as a focal or diffuse thickening and hyperenhancement of the bowel wall, along with the dilatation of the proximal bowel, and functional obstruction due to tumor infiltration of nerves in the peritoneum, which presents as extensive dilatation without focal irregular thickening and enhancement of the wall (25, 27). If one or more of these tracts presented with the abovementioned manifestations, a triple tract dilatation (TTD) sign was considered to be present. Notably, patients with other causes of dilatation (such as inflammatory disease and cholelithiasis) were considered to be absent from TTD sign.

The presence of extensive lymph node metastasis (ELM) included the enlargement of the lymph node around para-aortic (PAN) and around the celiac artery and its branches (bulky N) (28). PAN was determined when No. 16 lymph node had a short diameter larger than 10 mm. Bulky N was determined when the NO.8, 9, and 11 lymph nodes had a short diameter larger than 30 mm, or at least two adjacent lymph nodes with a short diameter of each larger than 15 mm (28). We recorded the presence of ELM that met the above criteria.

The grade of ascites was scaled as follows (29): grade 0 = ascites not detected by CT scan; grade 1 = ascites located only in the upper or lower abdominal cavity; grade 2 = neither grade 1 nor grade 3; grade 3 = ascites extending throughout the abdominal cavity.

### Statistical analysis

The statistical analysis was conducted using RStudio 3.5.0 (R Foundation for Statistical Computing, Vienna, Austria) and SPSS 20.0 (IBM, Armonk, USA). Continuous variables were presented as the median with interquartile ranges (IQR). Categorical variables were shown as numbers with percentages. The optimal cut-off value for the CT-PMS was determined using the X-tile 3.6.1 (Yale University School of Medicine, New Haven, CT, USA) to build the classified CT-PMS. Survival curves were calculated using Kaplan–Meier analysis and compared using log-rank test. Bonferroni correction was used to adjust *P* value for multiple comparisons. Univariable and multivariable Cox proportional hazards models were used to identify the prognostic factors for OS. Values with *P* <.10 on the univariate analysis were included in the multivariate analyses. The agreement between two radiologists was assessed using the kappa statistic for categorical variables and the weighted kappa statistic for ordinal categorical variables. The values of 0.81–1.00, 0.61–0.80, 0.41–0.60, 0.21–0.40, and <0.20 were indicated to be in almost perfect agreement, substantial agreement, moderate agreement, fair agreement, and slight agreement, respectively (30). A statistically significant difference was reported when *P* <.05 (two-sided).

## Results

### Patient characteristics

A total of 66 gastric cancer patients with synchronous PM were included in the present study. The median follow-up period was 319 days (IQR: 171–457 days). All patients had died by the time of study closure. The median age of these patients was 57 years (IQR: 48–64 years). Most of these patients had diffuse-type (*n* = 39, 59.10%) and poor differentiation (*n* = 48, 72.73%) on histopathology. Furthermore, all patients received palliative systemic chemotherapy, alone or in combination with supportive care. The clinicopathological characteristics of these patients are summarized in **Table 1**. The most commonly involved region was the greater omentum (*n* = 35, 53.00%), followed by the GCL (*n* = 31, 47.00%), lateral parietal peritoneum (*n* = 22, 33.30%), posterior parietal peritoneum (*n* = 19, 28.80%), mesentery (*n* = 17, 25.80%), GHL and HDL (*n* = 17, 25.80%), GSL (*n* = 16, 24.20%), pelvic parietal peritoneum (*n* = 12, 18.20%), superior parietal peritoneum (*n* = 6, 9.10%), and perihepatic visceral peritoneum (*n* = 2, 3.00%).

### Kaplan–Meier analysis

**Figure 4:**
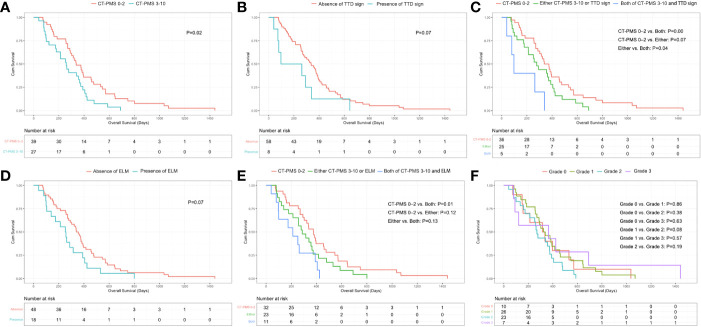
**(A)** Significant difference in OS between patients with CT-PMS values of 0–2 and 3–10 (P = .02, log-rank test) can be observed. **(B)** Borderline significant difference in the OS between patients with and without a triple tract dilatation (TTD) sign can be observed (P = .07, log-rank test). **(C)** Significant difference in OS between patients with both CT-PMS of 3–10 and the presence of triple tract dilatation sign and patients only with CT-PMS of 0–2 (P = .00, log-rank test, P value has been adjusted by Bonferroni correction and P <.02 [0.05/3] was considered statistically significant difference) can be observed. **(D)** Borderline significant difference in the OS between patients with and without ELM can be observed (P = .07, log-rank test). **(E)** Significant difference in OS between patients with both CT-PMS 3–10 and the presence of ELM and patients only with CT-PMS of 0–2 (P = .01, log-rank test, P value has been adjusted by Bonferroni correction and P <.02 [0.05/3] was considered statistically significant difference) can be observed. **(F)** T here were no significant differences in OS between patients with different grade of ascites (P >.00, log-rank test, P value has been adjusted by Bonferroni correction and P <.01 [0.05/6] was considered statistically significant difference) can be observed.

The optimal cut-off value for the CT-PMS was 2 by X-tile. There were 39 patients with a CT-PMS of 0–2 and 27 patients with a CT-PMS of 3–10. The median OS of patients with a CT-PMS of 0–2 was 338 days (IQR: 255–546 days), while that of the remaining patients was 258 days (IQR: 100–390 days). A significant difference in the OS was perceived between patients with CT-PMS of 0–2 and 3–10 (*P* = .02, log-rank test; **Figure 4A**).

Eight patients presented with TTD sign. Among these patients, three had biliary dilatation, three had bilateral urothelial dilatation, and the other two had bowel dilatation. The median OS of patients with the presence of TTD sign was 97 days (IQR: 73–292 days), while that of the remaining patients was 335 days (IQR: 186–460 days). A borderline significant difference in the OS was perceived between patients with and without TTD sign (P = .07, log-rank test; **Figure 4B**). Furthermore, we divided patients into three groups including five patients with both CT-PMS of 3–10 and the presence of TTD sign, 25 patients with either CT-PMS of 3–10 (n=22), or the presence of TTD sign (n=3) and 36 patients with only CT-PMS of 0–2. A significant difference in the OS was perceived between patients with CT-PMS of 0–2 and patients with both CT-PMS of 3–10 and the presence of TTD sign (P = .00, log-rank test; P value has been adjusted by Bonferroni correction and P <.02 [0.05/3] was considered statistically significant difference; **Figure 4C**).

Eighteen patients presented with ELM. Among these patients, ten patients had both PAN and Bulky N, five had Bulky N, and the other three had PAN. The median OS of patients with ELM was 252 days (IQR: 104–390 days), while that of the remaining patients was 340 days (IQR: 186–476 days). A borderline significant difference in the OS was perceived between patients with and without ELM (P = .07, log-rank test; **Figure 4D**). Furthermore, we divided patients into three groups including 11 patients with both CT-PMS of 3–10 and ELM, 23 patients with either CT-PMS of 3–10 (n=16), or ELM (n=7), and 32 patients with CT-PMS 0–2. A significant difference in the OS was perceived between patients with CTPMS of 0–2 and patients with both CT-PMS of 3–10 and ELM (P = .01, log-rank test; P value has been adjusted by Bonferroni correction and P <.02 [0.05/3] was considered statistically significant difference; **Figure 4E**).

There were 10 patients with ascites of grade 0, 26 patients with ascites of grade 1, 23 patients with ascites of grade 2, and seven patients with ascites of grade 3. There was no optimal cut-off value for the grade of ascites by X-tile. The median OS for patients with grade 0 ascites was 315 days (IQR: 157–531 days), for patients with grade 1 ascites was 323 days (IQR: 255–460 days), for patients with grade 2 ascites was 270 days (IQR: 161–390 days), while for the remaining patients with grade 3 ascites was 364 days (IQR: 91–690 days). There was no significant difference in the OS between patients with different grades of ascites (**Figure 4F**).

### Univariable and multivariable Cox analysis

In the univariate Cox analysis, the age, gender, ECOG PS, histology type, chemotherapy regimen, location of the primary tumor, Bormann type, and the grade of ascites did not have a prognostic impact (**Table 1**), while the CT-PMS and the classified CT-PMS (dichotomized by the optimal cut-off value of CT-PMS) were associated with the OS (hazard ratio [HR]: 1.10; 95% confidence interval [CI]: 1.00–1.21; *P* = .049; HR: 1.84; 95% CI: 1.10–3.07; *P* = .02). Differentiation, the presence of TTD sign, and the presence of ELM showed a predictive trend for OS, but failed to achieve statistical significance (HR: 1.77; 95% CI: 1.00–3.13; *P* = .05 for differentiation; HR: 1.97; 95% CI: 0.93–4.18; *P* = .08 for TTD sign; HR: 1.67; 95% CI: 0.96–2.90; *P* = .07 for ELM). In the multivariate analysis included CT-PMS (without the classified CT-PMS), after controlling for differentiation, TTD sign, and ELM, the CT-PMS was identified to be independently associated with survival (HR: 1.10; 95% CI: 1.00–1.21; *P* = .049). In the multivariate analysis included classified CT-PMS (without the CT-PMS), after controlling for differentiation, TTD sign, and ELM, the classified CT-PMS was identified to be independently associated with survival (HR: 1.84; 95% CI: 1.10–3.07; *P* = .02).

### Interobserver variability

The interobserver agreement for assessing PM signs of the individual regions varied from fair to substantial: *κ* = 0.62 (95% CI: 0.41–0.83) for the GSL, *κ* = 0.57 (95% CI: 0.34–0.81) for the GHL and HDL, *κ* = 0.64 (95% CI: 0.45–0.82) for the GCL, *κ* = 0.21 (95% CI: -0.18 to 0.61) for the perihepatic visceral peritoneum, *κ* = 0.74 (95% CI: 0.56–0.92) for the mesentery, *κ* = 0.70 (95% CI: 0.52–0.87) for the greater omentum, *κ* = 0.42 (95% CI: 0.09–0.75) for the superior parietal peritoneum, *κ* = 0.64 (95% CI: 0.44–0.84) for the posterior parietal peritoneum, *κ* = 0.60 (95% CI: 0.39–0.81) for the lateral parietal peritoneum, and *κ* = 0.58 (95% CI: 0.30–0.85) for the pelvic parietal peritoneum. Although the interobserver agreement was suboptimal in individual peritoneal regions, the summation of the involved regions compensated for the differences. The CT-PMS and the classified CT-PMS evaluated by the two radiologists had a substantial agreement (weighted kappa value [*κ*_w_] = 0.63 [95% CI: 0.54–0.72] for CT-PMS; *κ* = 0.77 [95% CI: 0.63–0.92] for the classified CT-PMS). The interobserver agreement for assessing tract dilatation varied from substantial to almost perfect: *κ* = 0.66(95% CI: 0.04–1.28) for bowel dilatation, *κ* = 0.65 (95% CI: 0.20–1.10) for bile dilatation, *κ* = 1.00 (95% CI: 1.00–1.00) for urothelial dilatation and *κ*_w_ = 0.77 (95% CI: 0.53–1.02) for the TTD sign. The interobserver agreement for assessing ELM was almost perfect: *κ* = 0.84 (95% CI: 0.68–0.99). The interobserver agreement for assessing ascites grade was substantial: *κ*_w_ = 0.79 (95% CI: 0.67–0.91).

## Discussion

The present study explored the CT characteristics to predict the prognosis of patients with gastric cancer and synchronous peritoneal metastasis. Among patients with peritoneal metastasis, patients with CT-PMS of 3–10 showed a decreased prognosis. Patients with either TTD sign or ELM had a tendency to a poor prognosis. Patients with both CT-PMS of 3–10 and TTD sign or both CT-PMS of 3–10 and ELM showed significantly shortened OS.

The CT procedures were tailored for better visualization of PM through the combination of window adjustment and multiplanar reconstruction. The optimal window width/level should be adjusted to clearly demonstrate the homogeneous granular background of fat tissues (22). Interactively reading the coronal and sagittal planes in addition to the mere axial plane can improve the visualization and localization of PM lesions (22). Some studies have demonstrated that the additional interpretation of coronal and sagittal planes could improve the detection of lesions on the perihepatic visceral peritoneum and superior parietal peritoneum, which run horizontally (12, 16, 22). The sagittal plane can provide a clear section view of the GCL extending to the transverse colon (16, 22, 31). One study also described the improvement in sensitivity from 64% in axial planes to 82% in MPR images during the PM diagnosis (12).

The PCI method divided the abdominal cavity into 9 regions based on two transverse and two sagittal planes and divided the small bowel into another 4 regions. The PCI is commonly used to quantify the tumor extension of the PM intraoperatively (7, 32). However, due to the low sensitivity for detecting peritoneal metastases on CT and the inconsistency of the peritoneal region during CT examination and surgery as a result of abdominal organ motion, there were several discrepancies between CT-PCI (the same dividing method with surgical PCI) and surgical PCI in gastric cancer (18, 33). The positive predictive value of the typical PM signs on CT ranged between 75% and 83%, indicating that the most of peritoneal regions with the aforementioned typical PM signs have peritoneal metastases (13, 14). Because the goal of our study was to predict prognosis rather than diagnose peritoneal metastasis, we utilized the typical PM signs as a reference to count the affected peritoneal regions. Similar to earlier findings that patients with a high PM burden had worse survival, our results showed that patients with CT-PMS of 3–10 had poor overall survival (3–6). The interobserver reproducibility for assessing the classified CT-PMS was substantial and acceptable. The segmentation method of the peritoneum has the propensity to move from crude to more accurate, and recent researches advised segmenting the peritoneum based on their anatomical route (11, 24, 25). According to the gastric cancer dissemination routes, we examined 10 peritoneal regions, which cover the majority of the abdominal and pelvic cavity (11, 22–25). This approach was resistant to organ movement, such as the gastrointestinal tract’s peristalsis and respiration-induced displacements. In addition, it potentially provides a more detailed mapping of peritoneal deposits on baseline CT, which outperforms the CT-PCI with only the information of equivocal location.

Because patients with malignant bowel obstruction (MBO) caused by advanced cancer may suffer from severe organ function damage, resulting in rapid deterioration of the physical condition and quality of life, the median OS of these patients were 80 days. (27, 34). A committee proposed clear clinical criteria for MBO to facilitate clinical management (27). However, a suitable radiologic definition is yet to be elucidated. In addition, bile duct obstruction and hydronephrosis due to metastasis infiltration are indicators of incomplete cytoreduction surgery and signify a dismal prognosis (25). In this study, the TTD sign on CT was defined as the obstruction of the intrahepatic bile duct and/or bilateral urinary tract and/or bowel, which is caused by cancer extending from the nearby peritoneum (25, 27). Compared with patients without TTD sign, patients with TTD sign demonstrated a trend of dismal survival, which was consistent with the previous research results (34). Moreover, patients with both CT-PMS of 3–10 and TTD sign showed an extremely unfavorable prognosis due to a higher peritoneal metastasis burden.

The survival rates of gastric cancer patients with extensive lymph node have been less satisfactory (35). In our study, patients with ELM demonstrated a trend toward a more unfavorable survival outcome compared to those without. Moreover, patients with both CT-PMS of 3–10 and ELM showed an extremely dismal prognosis, while patients without ELM had a relatively promising prognosis. Similarly, Sugarbaker PH et al. recently reported that the median overall survival was 67.9 months in colorectal cancer patients with positive peritoneal metastases and negative lymph node metastasis, compared to only 31.2 months in patients with both positive peritoneal and lymph node metastasis (36). This stratification may bring more information for the therapeutic regimen of gastric cancer.

The present study has several limitations. First, it was a single-center retrospective study that involved a limited number of patients. However, patients included in our study did not receive chemotherapy or any abdominal invasive operation before baseline CT, which could avoid the confounding factors induced by treatment. Further multicenter large-cohort prospective studies are needed to confirm the results of our study. Second, the metastasis deposit detection was mainly on the thick slice thickness images. However, a study showed that 5-mm slices and 1-mm slices had the same performance in detecting peritoneal deposition, and the 5-mm slices were enough for an adequate diagnosis of peritoneal carcinomatosis in most cases (12). Third, although the interobserver consistency for determining the metastasis in individual peritoneal regions was suboptimal, the summation of the involved regions can compensate for the differences, and the interobserver consistency was substantial for the CT-PMS and the classified CT-PMS. Fourth, it lacks a region-by-region corresponding histological gold standard owing to apparent ethical reasons.

## Conclusion

The CT-PMS is a promising indicator for the quantitative evaluation of the PM extent in gastric cancer. It can be used for prognosis prediction in gastric cancer patients with synchronous PM. A CT-PMS of 3–10 signifies a poor prognosis. For patients with CT-PMS of 3–10, the presence of either TTD sign or ELM may help screen out patients prone to extraordinarily poor prognosis and remind clinicians to be more cautious while determining the optimum treatment decision.

## Data availability statement

The raw data supporting the conclusions of this article will be made available by the authors, without undue reservation.

## Ethics statement

The studies involving human participants were reviewed and approved by Peking Cancer Hospital. The ethics committee waived the requirement of written informed consent for participation.

## Author contributions

JL, LC and XS contributed equally to this work and share first authorship. XZ and LT contributed equally to this work and share corresponding authorship. All authors contributed to the article and approved the submitted version.
